# Association Between Polymorphisms in Estrogen Receptor Genes and Depression in Women: A Meta-Analysis

**DOI:** 10.3389/fgene.2022.936296

**Published:** 2022-07-19

**Authors:** Cuifen Li, Manli Xie, Weiwei Wang, Yanyan Liu, Dan Liao, Jingwen Yin, Hao Huang

**Affiliations:** ^1^ Department of Gynecology, Songshan Lake Central Hospital of Dongguan City, Dongguan, China; ^2^ Department of Obstetrics and Gynecology, Qingxi Hospital of Dongguan City, Dongguan, China; ^3^ Department of Obstetrics and Gynecology, Affiliated Hospital of Guangdong Medical University, Zhanjiang, China; ^4^ Department of Psychiatry, Affiliated Hospital of Guangdong Medical University, Zhanjiang, China; ^5^ Affiliated Hospital of Guangdong Medical University, Zhanjiang, China

**Keywords:** ERα, Erβ, polymorphism, women, depression

## Abstract

**Objective:** It is suggested that estrogen receptors (ERs) might be associated with the disproportionate vulnerability of women to depressive episodes. Several variants in ER-alpha (ERα) and ER-beta (ERβ) have been linked to depression, but the results were not consistent. Hence, we conducted a meta-analysis to evaluate the association between ERα/ERβ and depression in a cohort of women.

**Methods:** A comprehensive literature search was performed in public databases. The genetic association between polymorphisms in Erα/ERβ and depression risk in a cohort of women was evaluated by odds ratios (ORs) and 95% confidence intervals (CIs). Cochran’s Q test and the I^2^ index were used to evaluate heterogeneity.

**Results:** In total, 10 studies and 4 SNPs (rs2234693, rs9340799, rs4986938, rs1256049) were included in our meta-analysis. rs2234693 genotype was significantly associated with the risk of depression in women by dominant model (CC + CT vs TT, OR = 1.30, 95% CI: 1.09–1.55, *p* = 0.0031), recessive model (CC vs CT + TT, OR = 1.64, 95% CI: 1.00–2.67, *p* = 0.0478), additive model (CC vs TT, OR = 1.93, 95% CI: 1.12–3.35, *p* = 0.0189) and allelic model (C vs T, OR = 1.24, 95% CI: 1.10–1.39, *p* = 0.0003). For rs9340799, the frequencies of risk genotypes according to the dominant (GG + GA vs AA, OR = 1.47, 95% CI = 1.10–1.98, *p* = 0.0096, I^2^ = 0%, *p* = 0.43) and allelic (G vs A, OR = 1.33, 95% CI: 1.04–1.69, *p* = 0.0236, I^2^ = 0%, *p* = 0.39) models were significantly lower in women with depression than in controls within the Asian subgroup. For rs1256049, risk genotypes were significantly more frequent in depressed subjects than in controls under the dominant model (AA+ GA vs GG, OR = 1.62, 95% CI: 1.19–2.21, *p* = 0.0024) and the allelic model (A vs G, OR = 1.35, 95% CI: 1.07–1.72, *p* = 0.012) after sensitivity analysis by omitting one study which induce the heterogeneity.

**Conclusions:** The current meta-analysis is the first and most comprehensive investigation of the association between ERs and depression in women, and the findings support the concept that ERs participate in the etiology of sex heterogeneity in depression.

## 1 Introduction

Depression is characterized by a persistent depressed mood and/or loss of pleasure in activities (Greenberg et al., 2012). There is a notable sex difference in the epidemiology of depressive episodes, with a higher prevalence in women than in men (Parker and Brotchie, 2010) from early life through the mid-50s, especially among women in the menopausal and postpartum periods (Blazer, 2003; Pearlstein, 2015; Sassarini, 2016). This observation indicates that the factors involved in the production and regulation of steroid hormones might be involved in the sex heterogeneity of depression.

There is a great deal of evidence that the sex heterogeneity of depression is largely due to estrogens and estrogen receptors (ERs) (Schuit et al., 2005; Sundermann et al., 2010; Nalvarte et al., 2021). Withdrawal of estrogens plays a key role in the onset of depression in animal models (Stoffel and Craft, 2004). Clinical evidence also indicates that women in the postpartum and menopausal periods show increased vulnerability to depression due to their drastically reduced estrogen levels (Sassarini, 2016). Hormone replacement therapy has been shown to have the benefit of preventing depression (Grigoriadis and Kennedy, 2002; Dwyer et al., 2020). Moreover, estrogen is involved in the regulation of multiple neural molecular processes and neurotransmitters that have been strongly implicated in affective disorders (Hwang et al., 2020); for example, processes and systems associated with estrogen include neurodevelopment, neurodegeneration, synaptic plasticity, neuroinflammation, dopamine signaling, the serotonin (5-HT) system and the hypothalamic–pituitary–adrenal (HPA) axis (Ostlund et al., 2003; Varshney et al., 2017; Baghel and Srivastava, 2020; Hwang et al., 2020; Brand et al., 2021; Varshney et al., 2021). Estrogen may play a key regulatory role in “windows of vulnerability” to depression in women (Schiller et al., 2015).

The cellular effects of estrogens are mainly due to the activation of two estrogen receptors, known as ER alpha (ERα) and ERβ (Heldring et al., 2007). These two receptors belong to class 1 of the superfamily of nuclear hormone receptors (Sundermann et al., 2010). Both ERα and ERβ are expressed throughout the brain (Enmark et al., 1997), especially in brain regions associated with core deficiencies in depression, such as cognitive function and emotion (González et al., 2007). ERα is predominantly expressed in the hypothalamus and amygdala, which indicates that this receptor may be involved in the regulation of autonomic function and emotional regulation. ERβ is predominantly expressed in the hippocampal formation and entorhinal cortex and thalamus, indicating that this receptor may modulate cognitive function, memory and motor functions (Osterlund and Hurd, 2001). Thus, there is evidence that ERs might underlie the neuropathology of psychiatric disorders such as depression.

To date, several variants in these two receptors have been associated with depression, especially in women; these variants include rs2234693, rs9340799, rs4986938 and TA repeat in ERα as well as rs1256049 and rs2077647, rs1271572 in Erβ (Tsai et al., 2003; Kim et al., 2010; Ryan et al., 2012; Pinsonneault et al., 2013; Zhang et al., 2014; Kang et al., 2015; Różycka et al., 2016; He et al., 2017; Zhang et al., 2017; Tan et al., 2018). However, such results have not been consistently reported in population-based studies. The generalizability of these results is limited by the small sample size, which may lead to insufficient statistical power to detect a true effect. It is necessary to combine sample sizes through a meta-analysis in order to more precisely identify the genetic association of ERα/ERβ variants with depression in women.

## 2 Materials and Methods

The present study was performed and prepared in accordance with the guidelines proposed by the Cochrane Collaboration in the Cochrane Handbook for Systematic Reviews of Interventions (http://www.cochranehandbook.org) and the Preferred Reporting Items for Systematic Reviews and Meta-Analyses (PRISMA) statement (Liberati et al., 2009).

### 2.1 Search Strategy and Inclusion/Exclusion Criteria

A literature search was performed in PubMed, Science Direct, Wiley Online Library, the Cochrane Library, Google Scholar, and the Chinese National Knowledge Infrastructure (CNKI) from inception to 2 March 2022. No limitations were imposed on publication year or language. The search terms used to identify studies were a combination of terms identifying the genes (‘ERα’, ‘ER1’, ‘ESRα’, ‘ESR1’, ‘estrogen receptor 1’ and ‘estrogen receptor alpha’ or ‘ERβ’, ‘ER2’, ‘ESRβ’, ‘ESR2’, ‘estrogen receptor 2’ and ‘estrogen receptor beta’) and terms identifying the phenotypes of interest (‘MDD’, ‘depression’). Studies that met the following criteria were included in the analysis: 1) the study was performed on adult women (≥18 years old) with a diagnosis of depression; 2) the study reported data from at least one independent sample; and 3) the study provided sufficient data to calculate the odds ratios (ORs) and their 95% confidence intervals (CIs). Studies were excluded if 1) they were performed on youth under 18 years old or on men, or 2) there was no control group. The reference lists of all primary studies were also reviewed. For multiple publications covering the same sample, the one with the most complete and recent report was included.

### 2.2 Data Extraction and Quality Assessment

Two reviewers independently extracted the data and study characteristics, including the following: first author name and publication year, study design, participant information (population, ethnicity, sample size, mean age, sex), diagnostic criteria, assessment, genotyping methods, genotype and allelic distribution.

### 2.3 Risk-Of-Bias Assessment

A quality assessment was performed according to the guidelines laid out in the HuGE Review Handbook (Little et al., 2008). We applied a checklist designed to assess the quality and risk of bias in human genomic epidemiological studies, as described in a previous study (Elwood et al., 2019). The checklist included the following items: a clearly stated objective and hypothesis, clear eligibility criteria for participants, information bias in genotyping, selection bias, information bias in assessment of environmental factors, information bias in assessment of depression, restrictions on the ethnicity of participants, replicable statistical methods, assessment of Hardy-Weinberg equilibrium (HWE), sufficient descriptive data, and stated genotype frequencies. The assessment was conducted by two reviewers, and discrepancies were resolved by consensus. The results of the assessment are shown in Table 1.

**TABLE 1:** Assessment of the risk of bias in the pooled studies.

Study	Year	Clearly stated objective and hypothesis	Clear eligibility criteria for participants	Information bias in genotyping	Selection bias	Information bias in assessment of environmental factors	Information bias in assessment of depression	Restrictions on the ethnicity of participants	Replicable statistical methods	Assessment of HWE	Sufficient descriptive data	Stated genotype frequencies
Tan et al	2018	Yes	Yes	QC	No	NA	No	Yes	Yes	Yes	Yes	Yes
He et al	2017	Yes	No	QC	No	NA	No	Yes	Yes	Yes	Yes	Yes
Zhang et al	2017	Yes	Yes	QC	No	NA	No	Yes	Yes	Yes	Yes	Yes
Różycka et al	2016	Yes	No	QC	No	NA	Without diagnostic criteria	Yes	Yes	Yes	Yes	Yes
Kang et al	2015	Yes	Yes	QC	No	NA	No	Yes	Yes	Yes	Yes	Yes
Zhang et al	2014	Yes	Yes	QC	No	NA	No	Yes	Yes	Yes	Yes	Yes
Pinsonneault et al	2013	Yes	Yes	QC	No	NA	No	No	Yes	Yes	Yes	Yes
Ryan et al	2012	Yes	Yes	QC	Women aged 70–77	NA	No	Yes	Yes	Yes	Yes	Yes
Kim et al	2010	Yes	Yes	QC	No	NA	No	Yes	Yes	Yes	Yes	Yes
Tsai et al	2003	Yes	Yes	QC	No	NA	No	Yes	Yes	Yes	Yes	Yes

QC, details given of quality control procedures; NA, not available; HWE, Hardy-Weinberg equilibrium.

### 2.4 Statistical Analysis

HWE was tested using Pearson’s chi-squared test. Four genetic models were used in the meta-analysis: the dominant model, the recessive model, the additive model and the allelic model. ORs with 95% CI were used to assess the different distributions of ER gene polymorphisms in the depression and control groups within this cohort of women. Cochran’s Q test and *I* ^2^ index based on a chi-square distribution were applied to estimate heterogeneity. *I* ^2^ ranges from 0 to 100%. Low, moderate, high and extreme heterogeneity correspond to0-25%, 25–50%, 50–75% and75-100%, respectively (Liberati et al., 2009). If the result of the Q test was *p* > 0.05 and I^2^ < 50%, the Mantel–Haenszel method was used to estimate the pooled odds ratio, assuming a fixed effects model. Otherwise, ORs were pooled according to a random-effects model (DerSimonian and Laird). Sensitivity analysis for the overall effect was performed by omitting one study at a time. Potential publication bias was evaluated by Begg’s funnel plots, Egger’s (Egger et al., 1997) and Peters’ (Peters et al., 2006) regression tests. The two-tailed *p* values were used in all analyses, and *p* < 0.05 was regarded as statistically significant. The statistical analyses were performed using the *meta* package in R version 3.6.2 (R Core Team, Vienna, Austria) and R Studio version 1.2.1 (Certified B Corporations, Boston, United States ).

## 3 Results

**FIGURE 1:**
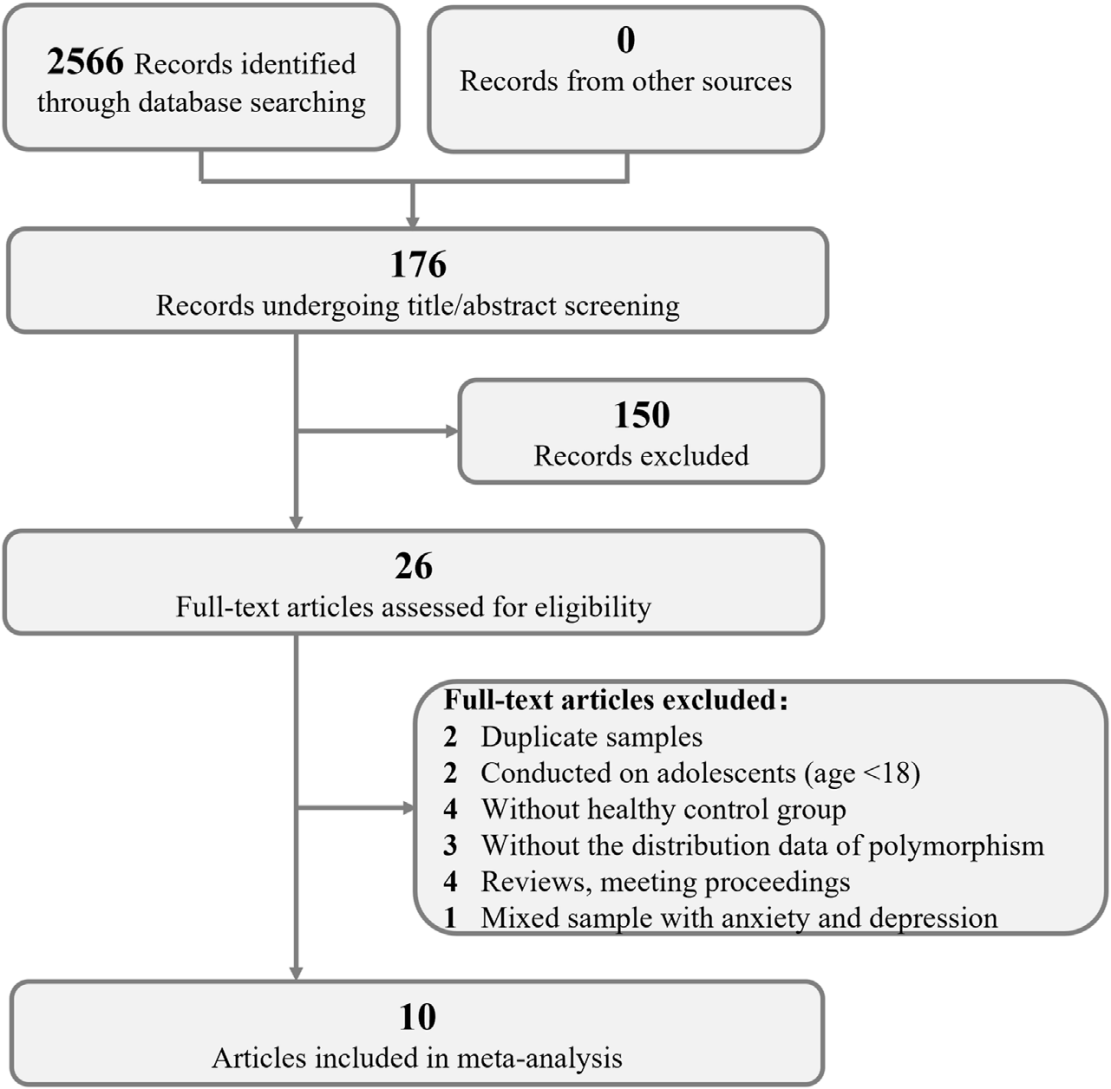
PRISMA flow chart of the inclusion and exclusion of studies. PRISMA = Preferred Reporting Items for Systematic Reviews and Meta-Analyses.

In total, 2566 articles were obtained from the initial search. The flow diagram of the process of selection is illustrated in Figure 1. A total of 176 studies were screened based on their titles, abstracts and contexts; 26 studies passed this screening. These 26 studies were evaluated by reading the full-text articles; at this stage, 15 studies were excluded because they were duplicate records, meeting proceedings, reviews or because they had insufficient data, no control groups or were conducted on adolescents. One study was excluded because it used a mixed sample of patients with menopausal anxiety and patients with depression (Zhou et al., 2012). Ultimately, data from 10 studies were included in our meta-analysis of the roles of ERα and ERβ in susceptibility to depression in women (Tsai et al., 2003; Kim et al., 2010; Ryan et al., 2012; Pinsonneault et al., 2013; Zhang et al., 2014; Kang et al., 2015; Różycka et al., 2016; He et al., 2017; Zhang et al., 2017; Tan et al., 2018). Three of these studies were conducted in Caucasians, and 7 were conducted in Asians. Data on 7 different SNPs were collected, four of which were reported in 3 independent samples and thus included in our meta-analysis (rs2234693, rs9340799, rs4986938, rs1256049). The data on rs2234693 in Kim’s (Kim et al., 2010) study and rs2077647 in Zhang’s (Zhang et al., 2014) study were excluded from the meta-analysis because the genotype distribution in the control group showed deviation in the HWE test. rs2077647 and rs1271572 in ERβ and TA repeat in ERα were not included in the meta-analysis due to the insufficient number of studies. The main characteristics of these studies are presented in Table 2.

**TABLE 2:** Characteristics of studies investigating polymorphisms of ERα and ERβ in women with depression.

Study	Year	Country	Ethnicity	Genotyped SNPs	Diagnostic criteria and assessment	Disease	Methods	HWE(*p*)
Tan et al	2018	Singapore	Asian	rs2234693, rs9340799, rs4986938, rs2077647, TA repeat	DSM-IV, EPDS	Perinatal depression	PCR-RFLP	>0.05
He et al	2017	China	Asian	rs2234693, rs9340799, rs4986938, rs1256049	SDS, HAMD, CCMD-3	Perinatal depression	PCR-PFLP	>0.05
Zhang et al	2017	China	Asian	rs4986938, rs1256049	DSM-IV, HAMD	Depression	PCR	>0.05
Różycka et al	2016	Poland	Caucasian	rs9340799	HAMD	Menopausal Depression	PCR-RFLP	>0.05
Kang et al	2015	China	Asian	rs4986938, rs1256049, rs1271572	BDI, DSM-IV	Perimenopausal depression	SNaPshot	>0.05
Zhang et al	2014	China	Asian	rs1256049, rs2077647	BDI, DSM-IV	Postpartum depression	PCR	<0.05 for rs2077647
Pinsonneault et al	2013	Canada	Caucasian (91%), with 2% Asian, 2% Hispanic and 4% other	rs2077647, TA repeat	DSM-IV, EPDS, MADRS	Postpartum depression	SNapShot,PCR-PFLP	>0.05
Ryan et al	2012	Frence	Caucasian	rs2234693, rs9340799	DSM-IV, CES-D	Menopausal depression	KASPar	>0.05
Kim et al	2010	Korean	Asian	rs2234693, rs9340799, rs4986938, rs1256049	DSM-IV, BDI	Post-menopausal depression	PCR-RFLP	<0.05 for rs2234693
Tsai et al	2003	China	Asian	rs2234693, rs9340799	DSM-IV, HAMD	Depression	PCR	>0.05

**FIGURE 2:**
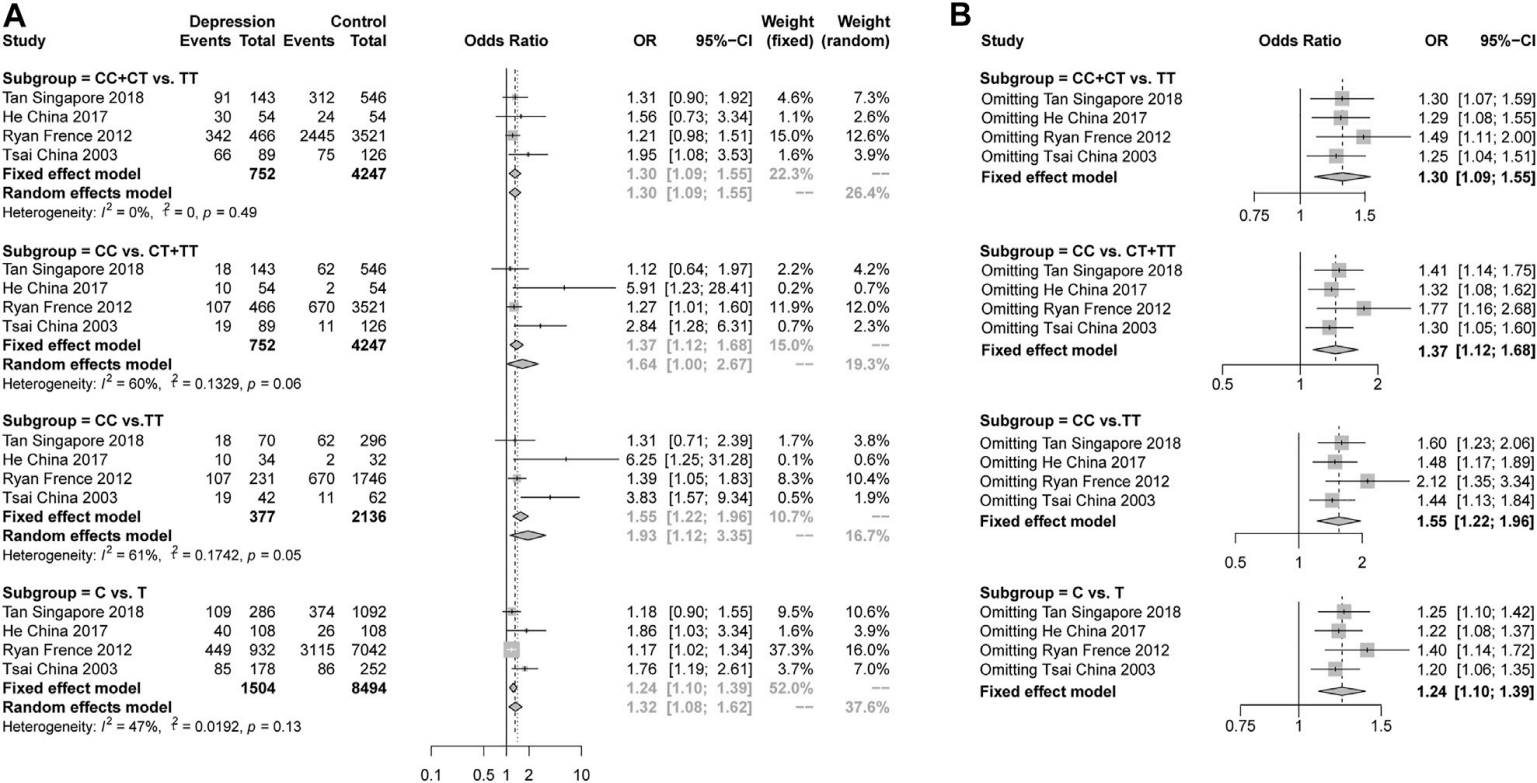
**(A)**, Forest plot of studies comparing the risk of the rs2234693 polymorphism of ERα between women with depression and controls; **(B)**, Meta-analysis with a fixed-effects model with individual studies omitted.

For rs2234693 in ERα, we collected 4 studies reporting the genotype and allelic distributions in 752 female depression patients and 4247 controls. A significant pooled OR was found in the dominant model (CC + CT vs TT, OR = 1.30, 95% CI: 1.09–1.55, *p* = 0.0031 under a fixed-effects model), the recessive model (CC vs CT + TT, OR = 1.64, 95% CI: 1.00–2.67, *p* = 0.0478 under a random-effects model), the additive model (CC vs TT, OR = 1.93, 95% CI: 1.12–3.35, *p* = 0.0189 under a random-effects model) and the allelic model (C vs T, OR = 1.24, 95% CI: 1.10–1.39, *p* = 0.0003 under a fixed-effects model) (Figure 2A). The heterogeneity analysis showed high heterogeneity in the recessive model (I^2^ = 60%, *p* = 0.06) and the additive model (I^2^ = 61%, *p* = 0.05). The sensitivity analysis was performed, and the data showed that no individual study qualitatively altered the pooled ORs in the four models (Figure 2B). This indicated the stability of the result. The shape of the funnel plots showed no publication bias (Supplementary Figure S4, Supplementary Table S1).

**FIGURE 3:**
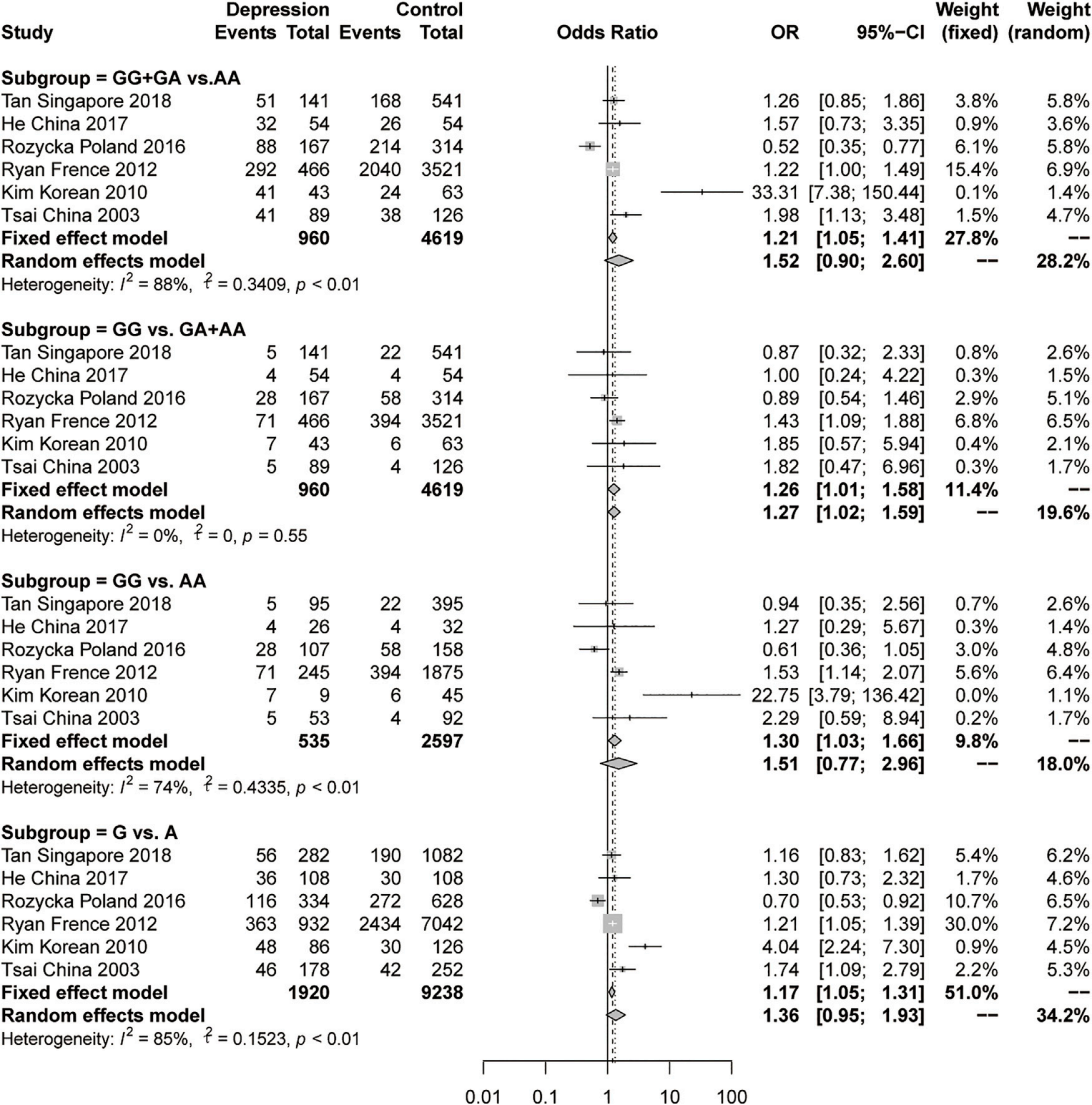
Forest plot of studies comparing the distribution of the rs9340799 polymorphism of ERα between women with depression and controls.

**FIGURE 4:**
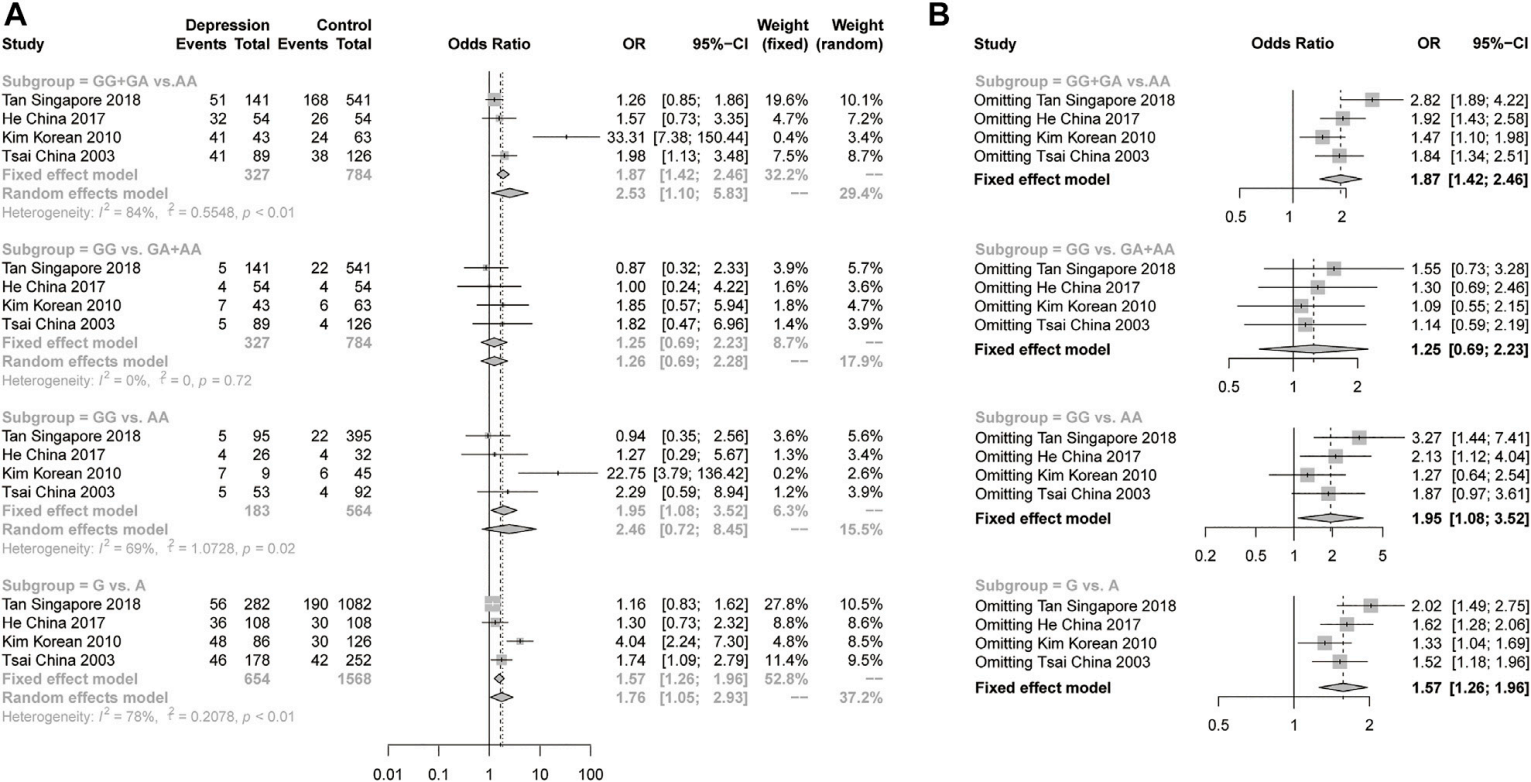
**(A)**, Forest plot of studies comparing the distribution of the rs9340799 polymorphism of ERα between women with depression and controls in the Asian subgroup. **(B)**, The sensitivity analysis of the studies pooled in the meta-analysis of rs9340799 polymorphism and women with depression in Asian subgroup by omitting one study at a time.

For the meta-analysis of rs9340799 in ERα, we collected 6 studies with 960 female depression patients and 4619 controls to calculate the pooled OR. In the results, a slightly significant difference was found in the recessive model (GG vs GA + AA) under a fixed-effects model (OR = 1.26, 95% CI: 1.01–1.58, *p* = 0.0405) with low heterogeneity (I^2^ = 0%, *p* = 0.55) (Figure 3). There was no significant difference and high heterogeneity shown in the dominant model, the additive model and the allelic model (Figure 3). Due to the high heterogeneity, the pooled sample was then evaluated using stratification analysis of ethnicity. In the Asian subgroup, there were 4 studies with 327 female depression patients and 784 controls pooled in the analysis. Significant differences were observed in the dominant model (GG + GA vs AA, OR = 2.53, 95% CI = 1.10–5.83, *p* = 0.0287, I^2^ = 84%, *p* < 0.01) and allelic model (G vs A, OR = 1.76, 95% CI = 1.05–2.93, *p* = 0.0307, I^2^ = 78%, *p* < 0.01) under the random-effects model, but still with a large heterogeneity (Figure 4A). The sensitivity analysis was then performed, and the results showed that omitting Kim’s study could dramatically decrease the heterogeneity to 0% in all four models. A significant difference persisted in the dominant models (GG + GA vs AA, OR = 1.47, 95% CI = 1.10–1.98, *p* = 0.0096, I^2^ = 0%, *p* = 0.43) and allelic model (G vs A, OR = 1.33, 95% CI: 1.04–1.69, *p* = 0.0236, I^2^ = 0%, *p* = 0.39) under the fixed-effect model, but the same was not true of the recessive model or the additive model (Figure 4B). There was no significant difference in the Caucasian subgroup, with a pooled sample size of 633 female depression patients and 3835 controls (Supplementary Figure S1).

**FIGURE 5:**
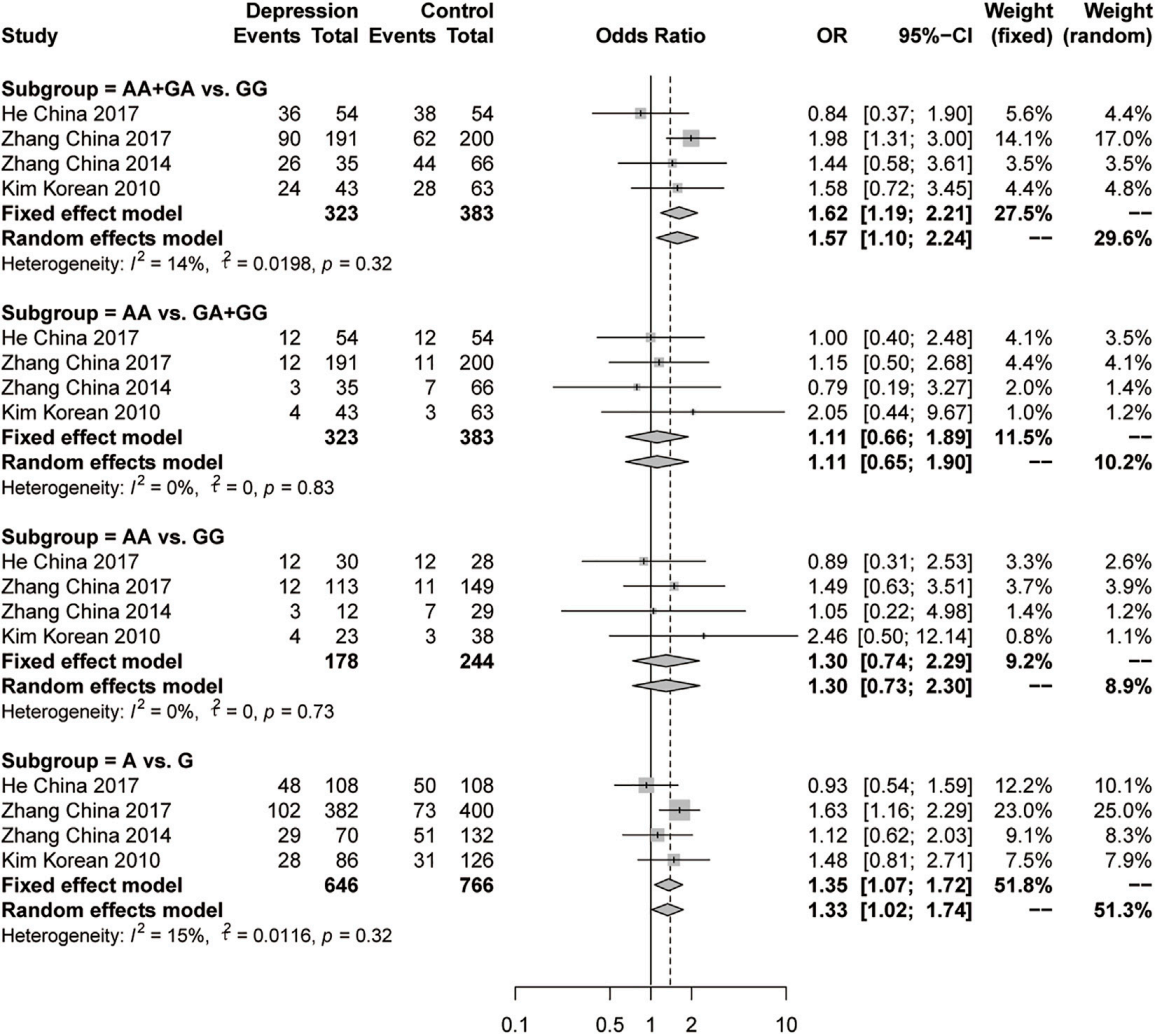
Forest plot of studies comparing the distribution of the rs1256049 polymorphism of ERβ between women with depression and controls after the omission of Kang’s study.

There were 5 studies genotyped rs1256049 in the ERβ gene in 388 female depression patients and 504 controls. There were no significant differences according to any of the four genetic models in the initial analysis (forest plots are shown in Supplementary Figure S2). Sensitivity analysis was performed to trace the sources of heterogeneity because of the high heterogeneity in the dominant model (AA+ GA vs GG, I^2^ = 59%, *p* = 0.05) and allelic model (A vs G, I^2^ = 56%, *p* = 0.06). The most effective reduction in heterogeneity (to 14% and 15%, respectively) emerged when Kang’s study was omitted (Kang et al., 2015). Then, significant differences were observed in the dominant model (AA+ GA vs GG, OR = 1.62, 95% CI: 1.19–2.21, *p* = 0.0024, fixed-effects model) and the allelic model (A vs G, OR = 1.35, 95% CI: 1.07–1.72, *p* = 0.012, fixed-effects model) (Figure 5). There was no significant difference in the recessive model or the additive model of rs1256049.

The meta-analysis failed to find an association between depression susceptibility and the rs4986938 polymorphism (Supplementary Figure S3) in women cohort. The shape of Begg’s funnel plots and the results of Egger’s (Egger et al., 1997) and Peters’ (Peters et al., 2006) regression tests showed no publication bias in any of the analyses (Supplementary Figure S4, Supplementary Figure S5, Supplementary Figure S6, Supplementary Figure S7, Supplementary Table S1).

## 4 Discussion

According to recent studies, ERα and ERβ polymorphisms are believed to be closely involved in sex-specific clinical symptoms and outcomes in women with depression (Hernández-Hernández et al., 2019). This suggests that investigation of ER genes and their functions might be important for understanding the pathophysiological mechanism of sex-specific depression risk. The present meta-analysis aggregated a large, population-based sample of female depression patients and controls to examine associations between polymorphisms in ER genes and sex-specific depression. A total of 10 case–control studies involving 1323 female depression patients and 5051 controls were pooled in the meta-analysis.

The rs2234693 polymorphism is located in the first intron of ERα and has been reported to impact the response of the ERα gene to estrogen by altering transcription factor binding (Herrington et al., 2002). Many reports have revealed that the rs2234693 polymorphism is a susceptibility factor for the onset of depression in women. The results from our meta-analysis provide evidence that rs2234693 of ERα was significantly associated with the risk of depression in women in four genetic models. There was a consistent tendency for the C allele of rs2234693 to be associated with an increased risk in women with depression in the pooled studies. Tsai et al. (Tsai et al., 2003) first reported the significant association of rs2234693 in female depression patients and was then identified in the Chinese population by He’s study (He et al., 2017). Tan’s (Tan et al., 2018) and Ryan’s (Ryan et al., 2012) studies also showed a higher frequency of the C allele in the depression group than in the control group. Moreover, a significant difference remained in the subgroup of Asians. The preponderance of the evidence confirms an association between the rs2234693 polymorphism and depression in women.

The ERα rs9340799 polymorphism has been widely studied, but conflicting results have been reported, and high heterogeneity was found in our meta-analysis. The variations and heterogeneity are particularly relevant in genetic heterogeneity in ethnicity. Therefore, subgroup analyses based on ethnicity were conducted in the present meta-analysis. In the Asian subgroup specifically, the G allele showed a tendency to be a risk factor for depression in women, and significant ORs were reported in the dominant model and the allelic model of rs9340799, albeit with high heterogeneity. The high heterogeneity might derive from the unstable allelic frequency, which was induced by the limitation of the sample size in individual studies. For instance, in the patient group of a Korean sample with 43 female depression patients and 63 controls, Kim et al. (Kim et al., 2010) noted a higher frequency of the G allele than other studies. Omitting Kim’s study could effectively reduce the heterogeneity (I^2^ = 0%). The results were still significant in the pooled sample from the three remaining studies. In the Caucasian subgroup, we failed to find any significant difference in the pooled data from two studies. Collectively, the G allele of the rs9340799 polymorphism is a potential risk allele for depression in Asian women.

rs1256049 is a silent mutation in exon 5 of ERβ gene. Zhang et al. (Zhang et al., 2017) reported T allele of rs1256049 was susceptible to major depressive disorder with a relatively larger sample from the Asian group. But the other studies failed to identify the results. The controversial results might drive by the limitation of the sample size which could lead to insufficient statistical power in detecting the difference. In our meta-analysis, significant differences were observed only in the dominant model and the allelic model. The sample size limitation also exited in the recessive model and the additive model of rs1256049. That is, the small sample size led to the extremely low frequency in the AA genotype in the recessive model and the additive model. The association between rs1256049 and depression needs to be identified in further study with larger sample size.

The limitations in this meta-analysis should be noted. For example, because of the limited effective number of samples, our meta-analysis was unable to test the relationship of ERα and ERβ polymorphisms with depression susceptibility in the Caucasian subgroup. Additionally, our meta-analysis pooled samples of women with depression in the gestational, menopausal, perinatal and other periods. Further subgroup analysis should be carried out based on these different periods marked by distinct hormonal fluctuations, such analysis may help to clarify the role of hormones in depression and contribute to effective treatment options.

## 5 Conclusion

In conclusion, our meta-analysis yielded evidence that the ERα polymorphism rs2234693 is associated with susceptibility to depression in women. Additionally, the ERα rs9340799 polymorphism is a potential risk factor for depression under the dominant and allelic models in Asian women. Further studies with larger sample sizes are still needed to support this conclusion.

## Data Availability

The original contributions presented in the study are included in the article/Supplementary Material, further inquiries can be directed to the corresponding authors. This study was registered in PROSPERO (registration no. CRD 42022324847).

## Abbreviations

DSM-IV, Diagnostic and Statistical Manual of Mental Disorders-4th edition; ICD, international classification of diseases; BDI, beck depression inventory; HAMD, hamilton rating scale for depression; SDS, Self-rating Depression Scale; CCMD-3, chinese classification and diagnostic criteria of mental disorders third edition; SCID, Structured Clinical Interview for DSM, disorders; EPDS, edinburgh postnatal depression scale; MADRS, Montgomery-Asberg Depression Rating Scale; MINI, international neuropsychiatric interview; HWE, Hardy–Weinberg equilibrium; CES-D, Centre for Epidemiology Studies–Depression Scale; PCR, polymerase chain reaction; RFLP: Restriction Fragment Length Polymorphism. KASPar, KBioscience’s competitive allele-specific PCR, amplification of target sequences and endpoint fluorescence genotyping.
