# Supplementary material for: Association Between Polymorphisms in Estrogen Receptor Genes and Depression in Women: A Meta-Analysis
Source: Front Genet. 2022 Jul 19;13:936296. doi: 10.3389/fgene.2022.936296 (PMC9343944; doi:10.3389/fgene.2022.936296)
Supplement: Supplementary file 1 [file DataSheet1.docx]

Supplementary Material

**Table S1**. Egger test and Peter test for funnel plot asymmetries of Polymorphisms

| Polymorphism | Genetic model | Number of studies | *P*_Egger_ | *P*_Peters_ |
| --- | --- | --- | --- | --- |
| rs2234693 | Dominant | 4 | 0.14 | 0.19 |
|  | Recessive | 4 | 0.22 | 0.08 |
|  | Additive | 4 | 0.17 | 0.06 |
|  | Allelic | 4 | 0.13 | 0.08 |
| rs9340799 | Dominant | 6 | 0.39 | 0.26 |
|  | Recessive | 6 | 0.74 | 0.74 |
|  | Additive | 6 | 0.57 | 0.44 |
|  | Allelic | 6 | 0.46 | 0.41 |
| rs1256049 | Dominant | 5 | 0.38 | 0.48 |
|  | Recessive | 5 | 0.62 | 0.92 |
|  | Additive | 5 | 0.68 | 0.85 |
|  | Allelic | 5 | 0.51 | 0.52 |
| rs4986938 | Dominant | 5 | 0.2 | 0.61 |
|  | Recessive | 5 | 0.05 | 0.64 |
|  | Additive | 5 | 0.06 | 0.65 |
|  | Allelic | 5 | 0.19 | 0.32 |


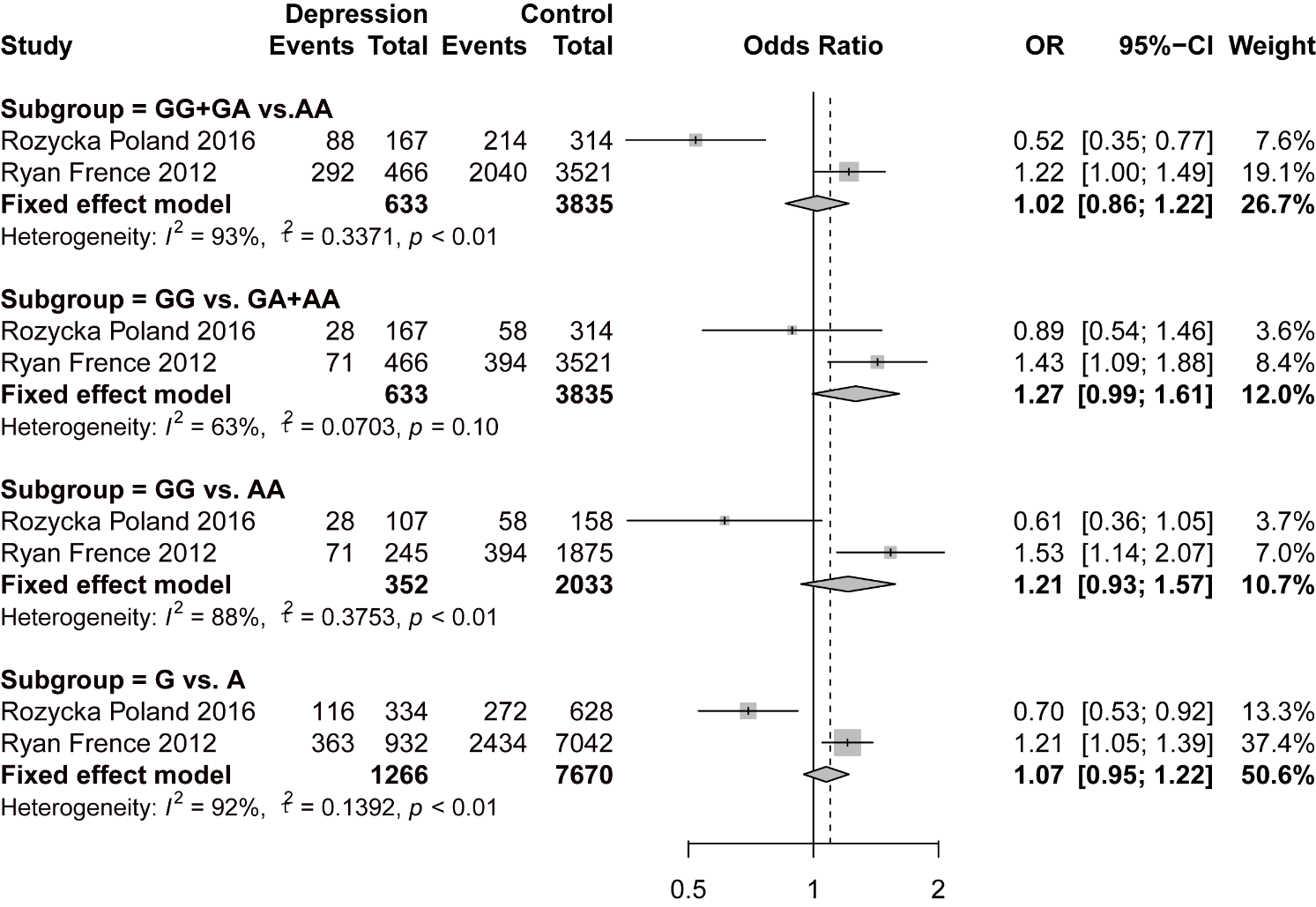


**Figure S1.** Forest plot of studies comparing the distribution of rs9340799 polymorphism of ERα between women with depression and controls in Caucasian subgroup.


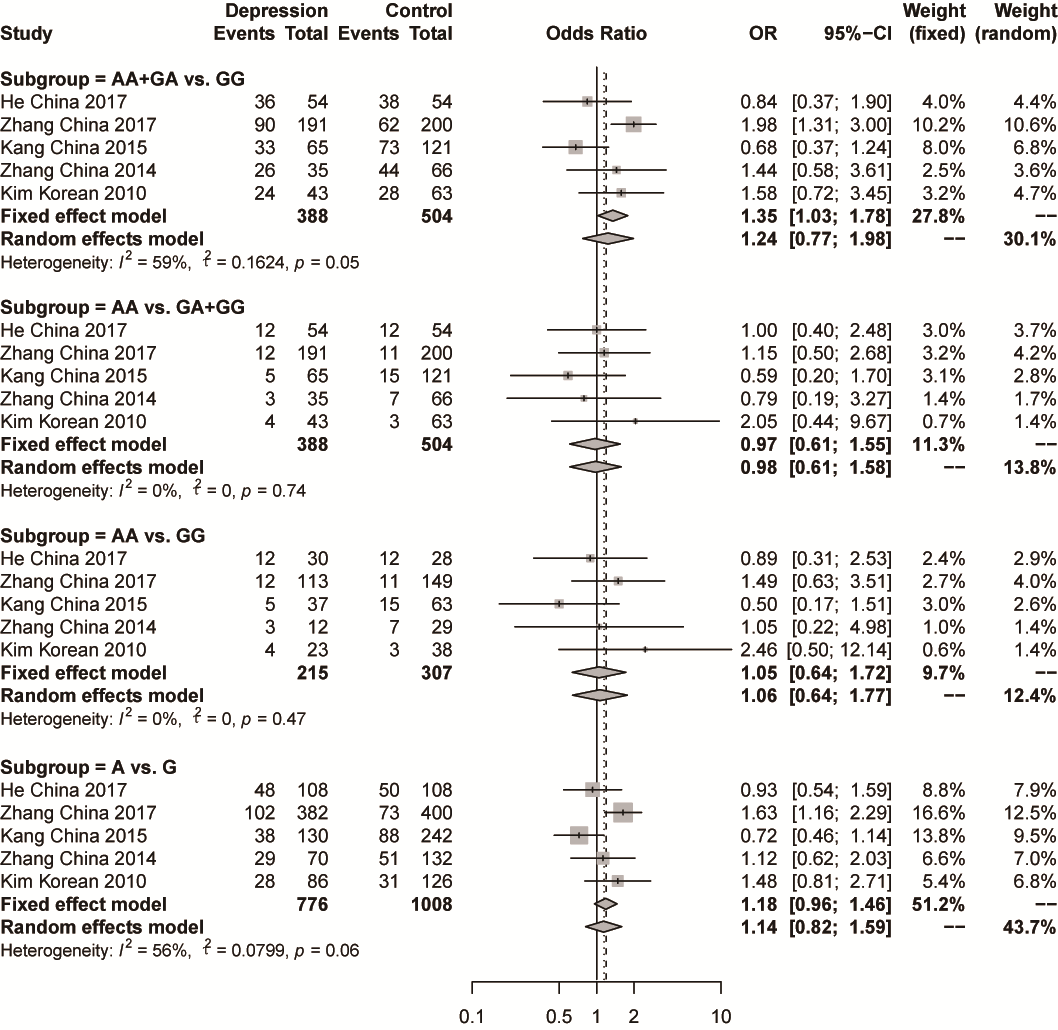


**Figure S2.** Forest plot of studies comparing the distribution of rs1256049 polymorphism of ERβ between women with depression and controls.


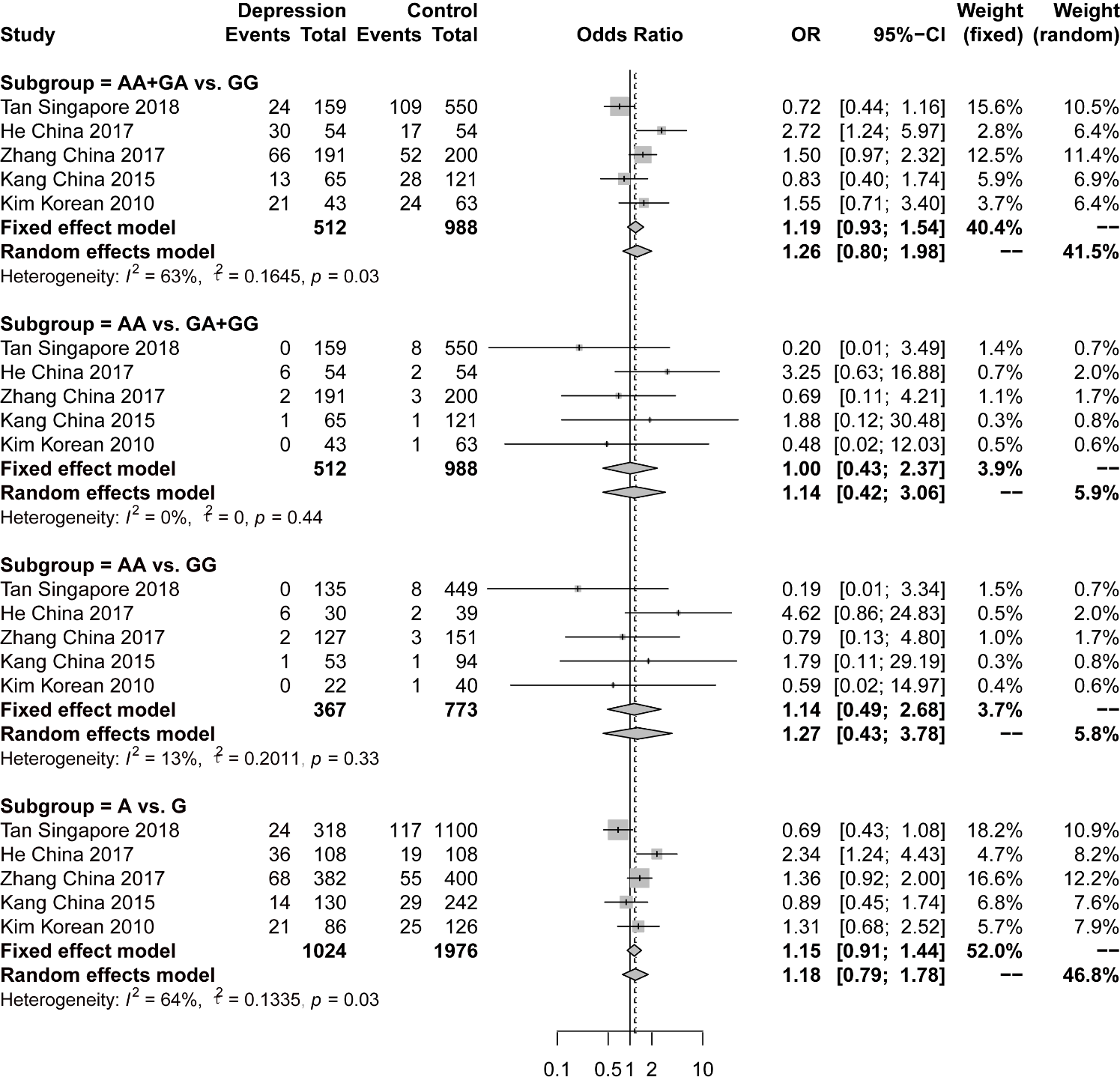


**Figure S3.** Forest plot of studies comparing the distribution of rs4986938 polymorphism of ERβ between women with depression and controls.


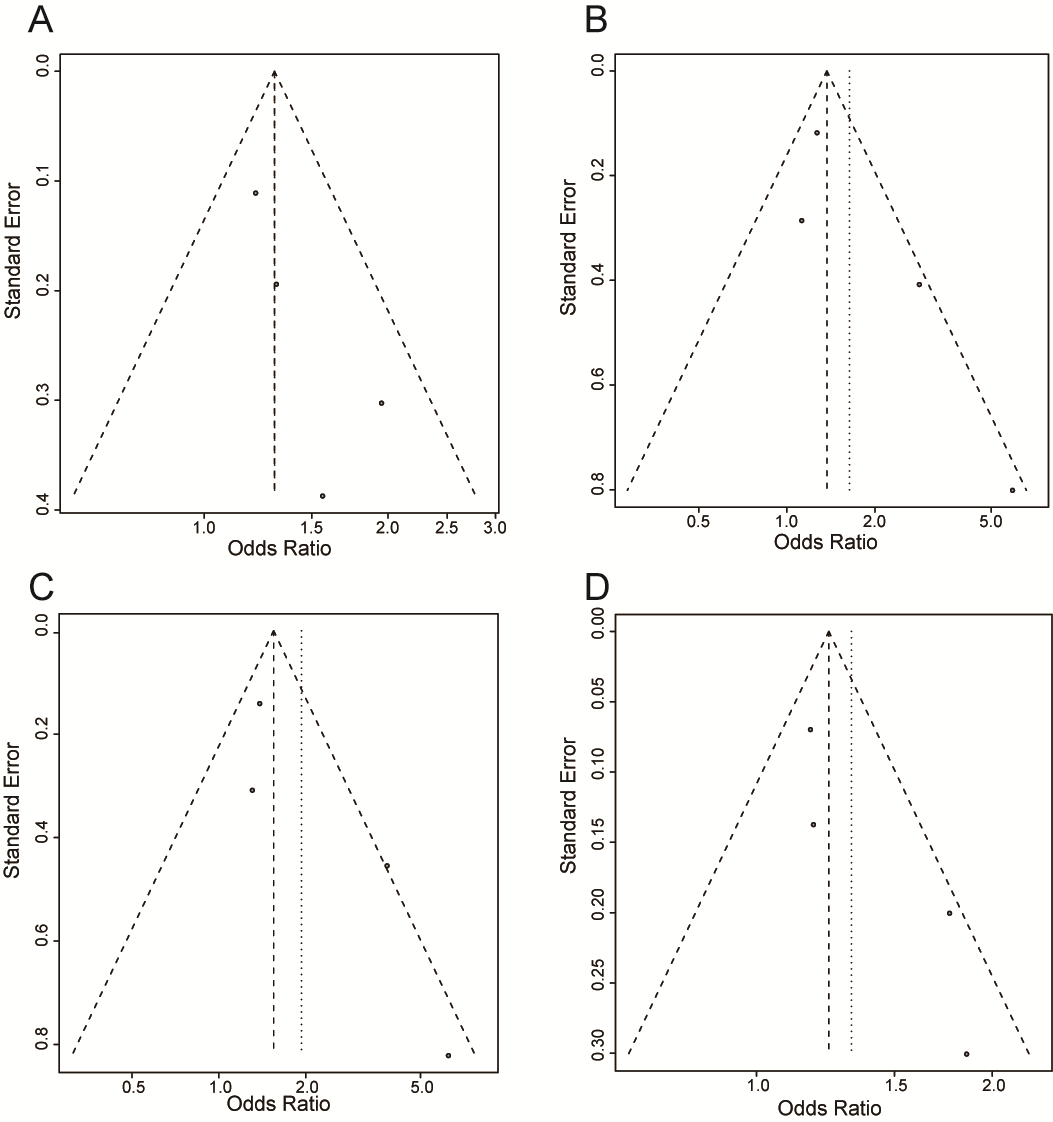


**Figure S4.** The Begg funnel plot for publication bias of literatures of rs2234693. A, Dominant model; B, Recessive model; C, Additive model; D, Allelic model.


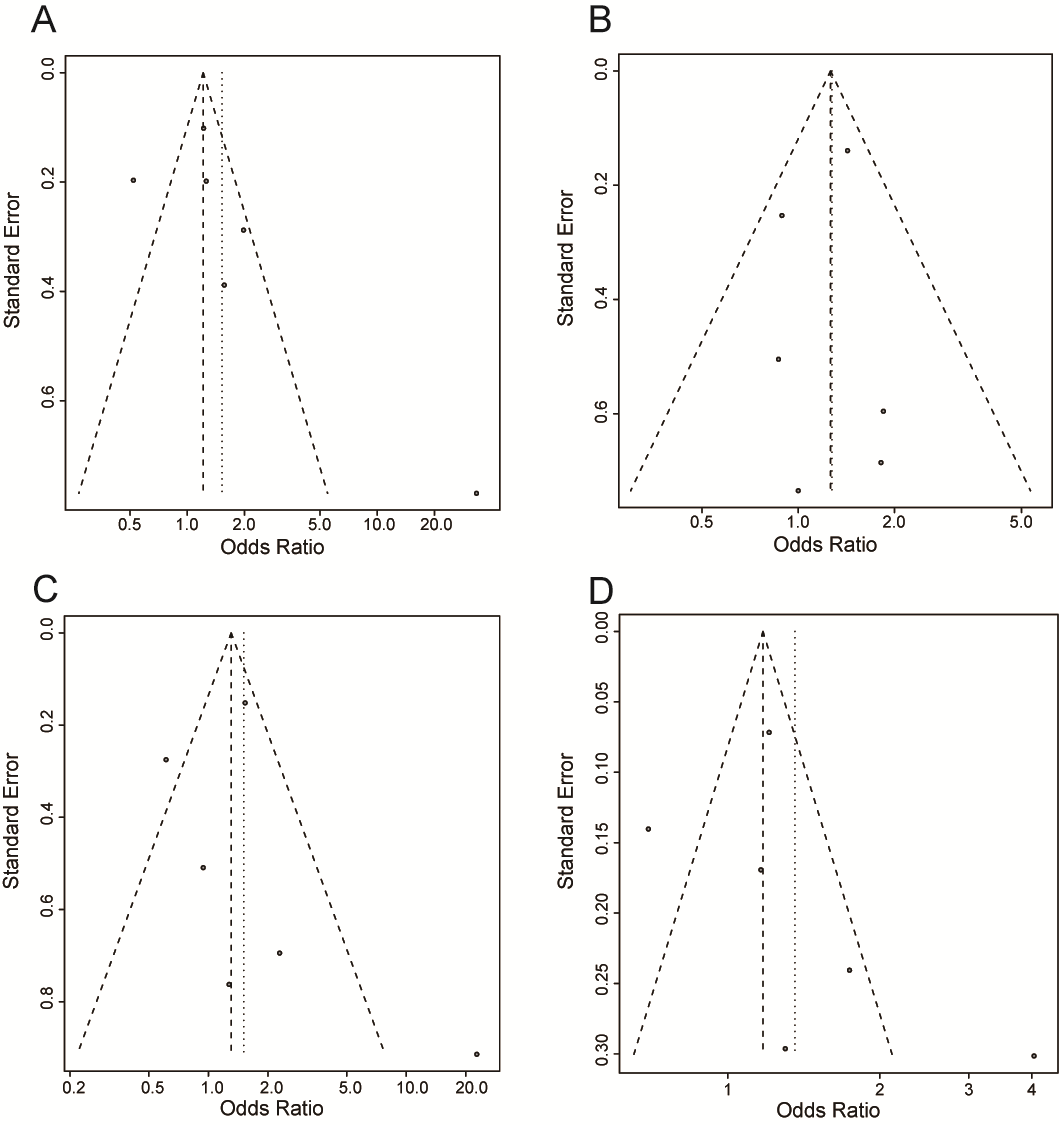


**Figure S5.** The Begg funnel plot for publication bias of literatures of rs9340799. A, Dominant model; B, Recessive model; C, Additive model; D, Allelic model.


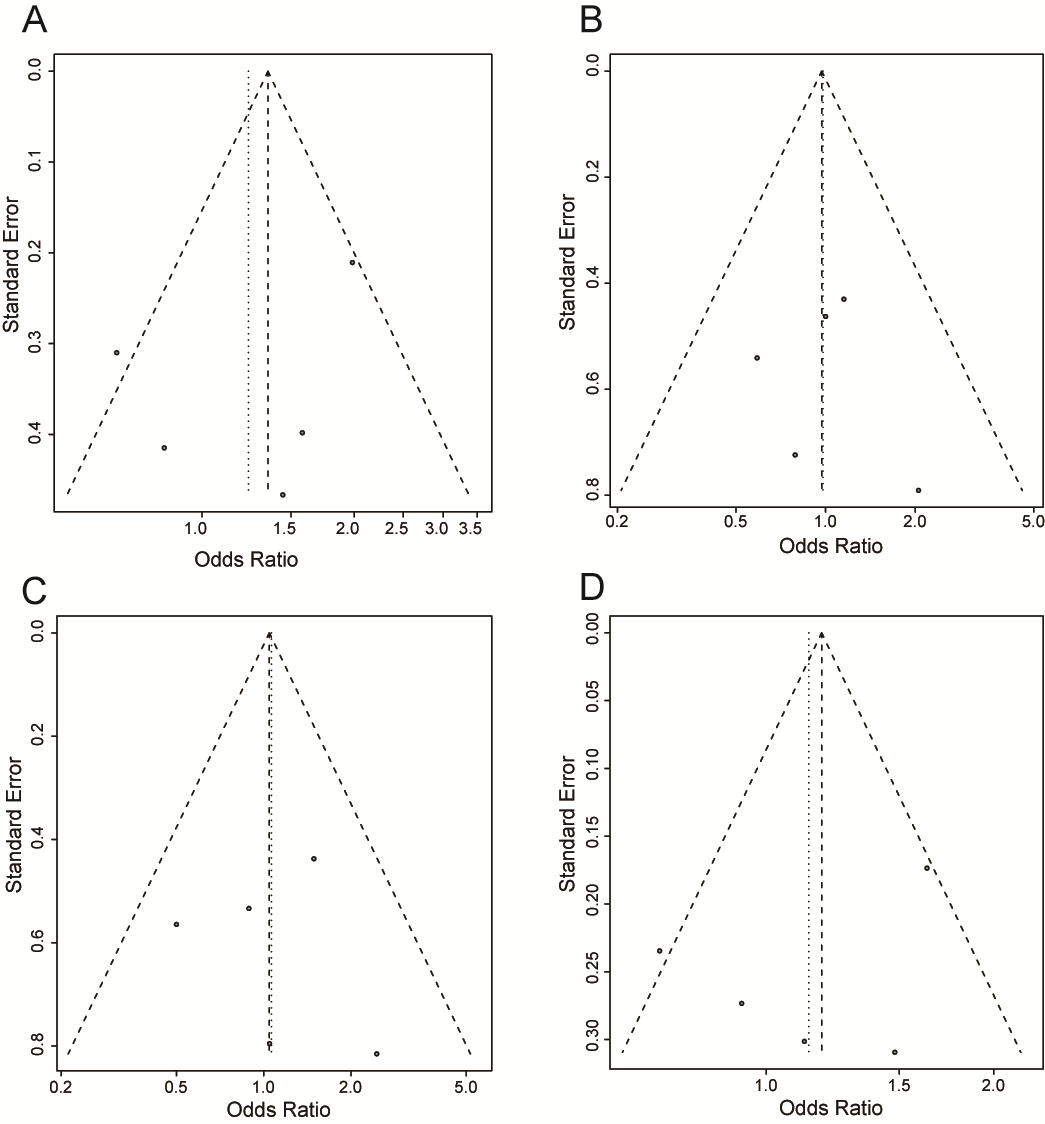


**Figure S6.** The Begg funnel plot for publication bias of literatures of rs1256049. A, Dominant model; B, Recessive model; C, Additive model; D, Allelic model.


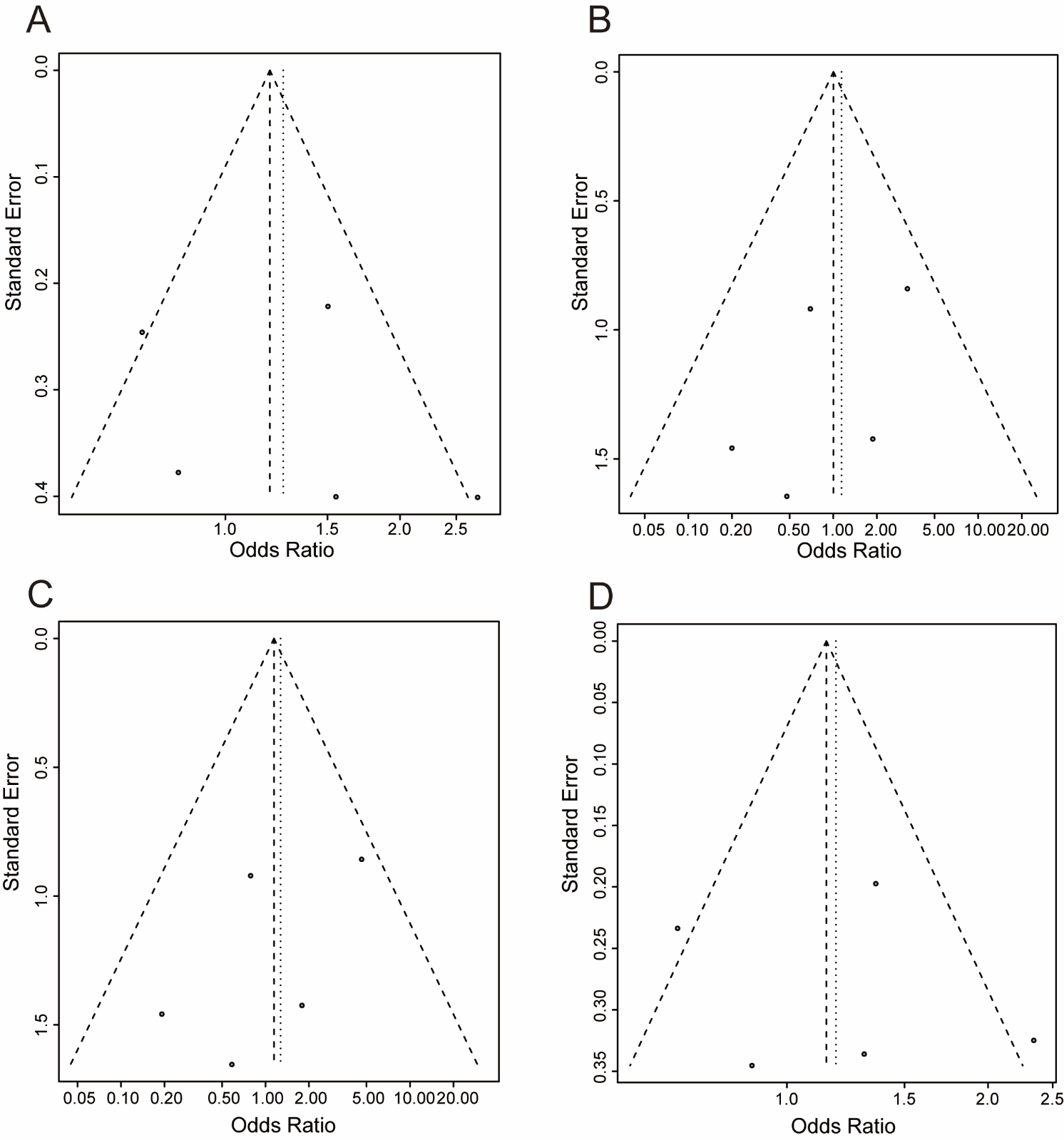


**Figure S7.** The Begg funnel plot for publication bias of literatures of rs4986938. A, Dominant model; B, Recessive model; C, Additive model; D, Allelic model.
